# Amphiphilic Block Copolymer PCL-PEG-PCL as Stationary Phase for Capillary Gas Chromatographic Separations

**DOI:** 10.3390/molecules24173158

**Published:** 2019-08-30

**Authors:** Tao Sun, Xiaomin Shuai, Kaixin Ren, Xingxing Jiang, Yujie Chen, Xinyu Zhao, Qianqian Song, Shaoqiang Hu, Zhiqiang Cai

**Affiliations:** 1 College of Chemistry and Chemical Engineering, Henan Key Laboratory of Function-Oriented Porous Materials, Luoyang Normal University, Luoyang 471934, China; 2 Liaoning Province Engineering Research Center for Fine Chemical Engineering of Aromatics Downstream, School of Petrochemical Engineering, Shenyang University of Technology, Liaoyang 111003, China

**Keywords:** capillary gas chromatography, stationary phase, amphiphilic block copolymer, separation performance, isomers

## Abstract

This work presents the first example of utilization of amphiphilic block copolymer PCL-PEG-PCL as a stationary phase for capillary gas chromatographic (GC) separations. The PCL-PEG-PCL capillary column fabricated by static coating provides a high column efficiency of 3951 plates/m for *n*-dodecane at 120 °C. McReynolds constants and Abraham system constants were also determined in order to evaluate the polarity and possible molecular interactions of the PCL-PEG-PCL stationary phase. Its selectivity and resolving capability were investigated by using a complex mixture covering analytes of diverse types and positional, structural, and cis-/trans-isomers. Impressively, it exhibits high resolution performance for aliphatic and aromatic isomers with diverse polarity, including those critical isomers such as butanol, dichlorobenzene, dimethylnaphthalene, xylenol, dichlorobenzaldehyde, and toluidine. Moreover, it was applied for the determination of isomer impurities in real samples, suggesting its potential for practical use. The superior separation performance demonstrates the potential of PCL-PEG-PCL and related block copolymers as stationary phases in GC and other separation technologies.

## 1. Introduction

Capillary gas chromatography (GC) is one of efficient separation techniques in petrochemical engineering, environmental protection, food analysis, and biological medicine because of its inherent advantages of high resolving capability and selectivity, fast analysis speed, as well as low cost [1,2,3,4]. As the core component in GC system, a stationary phase with high selectivity enables effective separation of analytes with close nature. In the past decades, diverse types of GC stationary phases have been explored, such as polymeric materials, ionic liquids, macrocyclic compounds and metal organic frameworks, etc., [5,6,7,8,9,10,11,12,13,14]. Polymeric materials with good solubility and film-forming ability, wide operational temperature range, and high chemical/thermal stability are advantageous as stationary phase in GC separations. Until now, siloxane polymers as stationary phases are most widely used in GC [15,16]. A stationary phase with high selectivity for analytes of varying nature is ideal for the analysis of complex samples. Modifying the side chains of polymer by different organic molecules is a common strategy to fine tune the polarity and selectivity of stationary phases [16,17,18]. Herein, we present a new strategy for the preparation and application of amphiphilic block copolymer as highly selective GC stationary phase.

Poly(*ε*-caprolactone) (PCL) is a saturated aliphatic polyester that is preferentially synthesized by ring-opening polymerization of *ε*-caprolactone [19]. Its repeating unit contains one relatively polar ester group and five nonpolar methylene groups [20]. It is well known that PCL is a hydrophobic and semi-crystalline polymer [21]. Its glass and melting temperatures are about −60 °C and 55 °C, respectively. Moreover, PCL possesses good solubility in common organic solvents, good film-forming ability and chemical/thermal stability [22,23]. Poly(ethylene)glycol (PEG) is a well-known hydrophilic polymer. It is a polyether composed of repeated ethylene glycol units [-(CH_2_CH_2_O)_n_] [24,25]. Moreover, it can be used as the hydrophilic part of amphiphilic block copolymers. Besides hydrophobic interaction, PEG moieties can provide some other interactions such as H-bonding and dipole–dipole interactions. The amphiphilic triblock copolymer of PCL-PEG-PCL can be simply synthesized via the ring-opening polymerization of *ε*-caprolactone in the presence of PEG [26,27,28]. The amphiphilic structure provides multiple molecular interactions for compounds of diverse types. The unique structure and favorable physicochemical features offer its good potential in separation science, which interested us to explore its separation performance as a GC stationary phase.

**Scheme 1:**
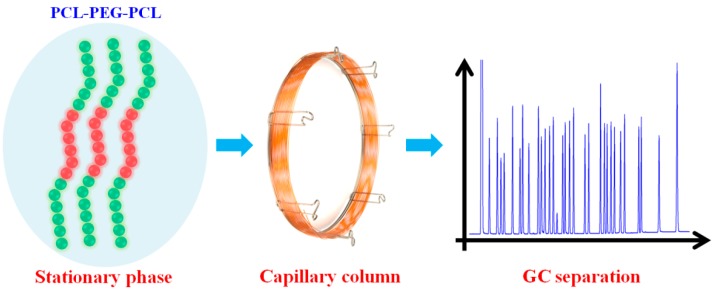
The PCL-PEG-PCL capillary column for gas chromatographic (GC) separation.

Herein, we present the report of using PCL-PEG-PCL as a stationary phase for GC separations (Scheme 1). The synthesized PCL-PEG-PCL was statically coated onto fused-silica capillary column. Then, the column efficiency, polarity and Abraham solvation system constants were determined. Afterward, the selectivity and resolving capability were investigated by utilizing the mixtures of diverse analytes and isomers. Moreover, the prepared PCL-PEG-PCL column was applied for the detection of impurity isomers in real samples. To our knowledge, this is the first example of employing amphiphilic block copolymer in chromatography. 

## 2. Results and Discussion

### 2.1. Characterization of the PCL-PEG-PCL

**Figure 1:**
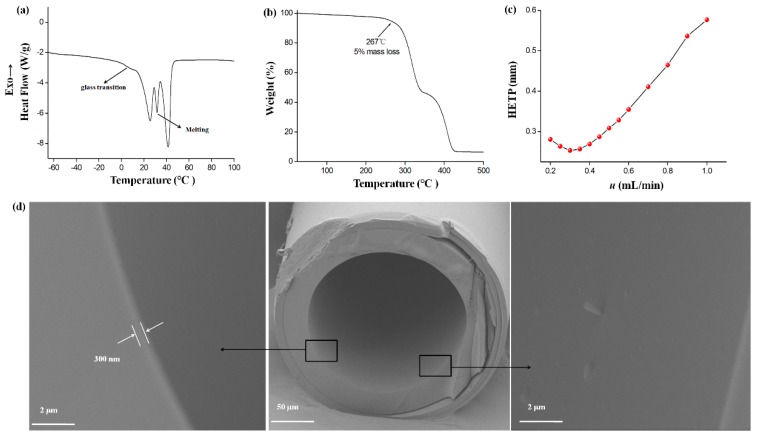
(**a**) Differential scanning calorimetry (DSC) curves of the PCL-PEG-PCL; (**b**) Thermogravimetric analysis (TGA) curve of the PCL-PEG-PCL; (**c**) Golay curve of the PCL-PEG-PCL column determined by *n*-dodecane at 120 °C; (**d**) the cross-section scanning electron microscopy (SEM) images on the inner wall surface and the coating thickness of the PCL-PEG-PCL column.

FT-IR and ^1^H-NMR were used to confirm the structure of PCL-PEG-PCL which was in good agreement with the literature [26,29,30]. The FT-IR spectrum of PCL-PEG-PCL is displayed in Figure S1. The absorption bands at 2942 cm^−1^ and 2864 cm^−1^ are attributed to C–H stretching vibrations of CH_2_ units of PCL chain. The strong band at 1721 cm^−1^ indicates the presence of ester carbonyl groups. The absorption bands at 1103 cm^−1^ and 1044 cm^−1^ are due to the C–O–C stretching vibration of the CH2–O–CH2 units of PEG block and the –COO– stretching vibrations, respectively. The ^1^H-NMR spectrum of the PCL-PEG-PCL is presented in Figure S2. The signals at 1.38, 1.72, 2.33, and 4.06 ppm are attributed to the methylenes of –(CH_2_)_3_–, –OCCH_2_–, and –CH_2_OOC– in PCL blocks, respectively. The sharp peak at 3.64 ppm is assigned to the methylene protons of the PEG –CH_2_CH_2_O– units, and the very weak peaks at 4.25 is attributed to the methylene protons of –CH_2_CH_2_O– that are linked to PCL blocks. The ^1^H-NMR spectra were used to calculate the PEG/PCL ratio according to the above literatures and the PEG/PCL blocks ratio was 1.5:1. In addition, the glass transition temperature (*T*_g_) of the synthesized copolymer was tested by differential scanning calorimetry (DSC). As shown in Figure 1a, the glass transition temperature of PCL-PEG-PCL was observed at approximately 2 °C. Thermogravimetric analysis (TGA) was employed to evaluate the intrinsic thermal stability of PCL-PEG-PCL in Figure 1b. The TGA curve showed that the PCL-PEG-PCL is stable up to 267 °C, thereby suggesting its low glass transition temperature and good thermal stability and feasibility as the stationary phase for GC separation.

### 2.2. Column Efficiency and Golay Plot

To evaluate the overall efficiency of the PCL-PEG-PCL column, its Golay curve was determined by measuring the height equivalent to a theoretical plate (HETP) of *n*-dodecane at different flow rates at 120 °C.As shown in Figure 1a, it attained the minimum HETP of 0.25 mm at 0.3 mL/min, corresponding to the column efficiency of 3951 plates/m. The high column efficiency can be attributed to the good solubility of PCL-PEG-PCL stationary phase in the solvent for column fabrication, facilitating its uniform coating on the capillary wall. In addition, Figure 1d presents the SEM cross-section images of the PCL-PEG-PCL column, confirming its uniform coating with the thickness of approximately 300 nm on the capillary column.

### 2.3. McReynolds Constants and Polarity

The polarity of PCL-PEG-PCL stationary phase was evaluated by McReynolds constants of the five probe solutes, i.e., benzene (X’), 1-butanol (Y’), 2-pentanone (Z’), 1-nitropropane (U’), and pyridine (S’), and the results are provided in Table 1 [31,32].As can be seen, the PCL-PEG-PCL stationary phase has an average value of 351, suggesting its moderate polarity. Additionally, the Abraham system constants of the PCL-PEG-PCL stationary phase were determined at three temperatures (80 °C, 100 °C, and 120 °C) and the results are provided in Table 2 [33,34,35]. Observably, the major interactions of the PCL-PEG-PCL stationary phase was H-bonding basicity (*a*), dipole–dipole (*s*),and dispersion interactions (*l*), which agree well with the results of McReynolds constants. Table S1 in the Supporting information lists the solutes and their solute descriptors.

### 2.4. Separation of a Complex Mixture of 30 Analytes of Diverse Types

**Figure 2:**
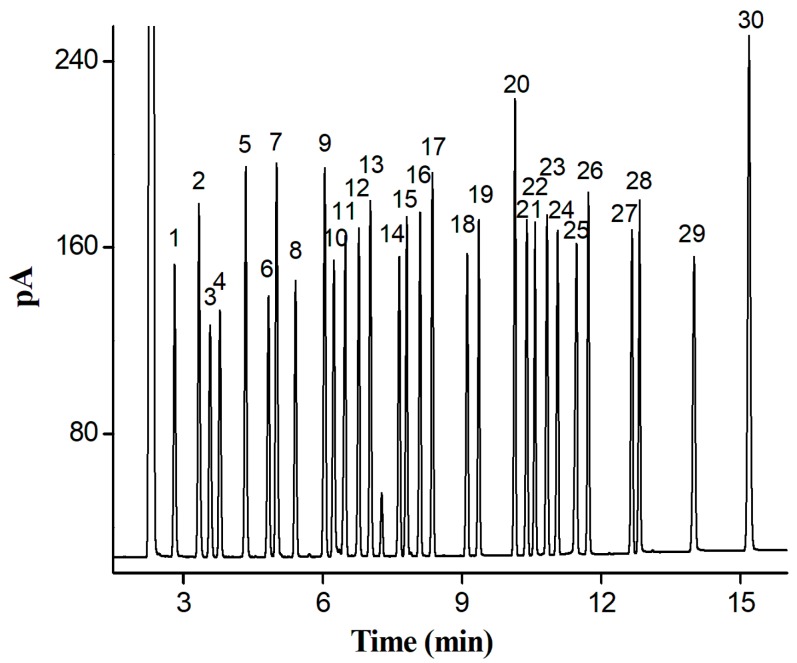
Separations of the complex mixture of 30 analytes of diverse types on the PCL-PEG-PCL column. Peaks: (1) *n*-nonane, (2) toluene, (3) 1-bromopentane, (4) 2-hexanone, (5) ethylenzene, (6) 1-bromohexane, (7) 2-propylbenzene, (8) 1-pentanol, (9) *sec*-butylbenzene, (10) 1-bromoheptane, (11) *n*-dodecane, (12) 1-hexanol, (13) 1,2,3-trimethylbenzene, (14) 1-bromooctane, (15) 2-nonanone, (16) 1-heptanol, (17) 1,2-dichlorobenzene, (18) methyl nonanoate, (19) 1-octanol, (20) acetophenone, (21) 2-undecanone, (22) 1-nonanol, (23) 1,3-dibromobenzene, (24) 1,2,3-trichlorobenzene, (25) *n*-hexadecane, (26) 1-undecanol, (27) methyl laurate, (28) 1-undecanol, (29) 1-dodecanol, (30) 2,3-dimethylphenol. Temperature program: 40 °C (1 min) to 160 °C at 10 °C/min, flow rate at 0.6 mL/min.

To comprehensively evaluate the separation performance of the PCL-PEG-PCL column for analytes of diverse varieties, a complex mixture of 30 analytes of diverse types, including *n*-alkanes, esters, bromoalkanes, ketones, alcohols, alkylbenzenes, halogenated benzenes, and xylenol, was employed. Its separations on the PCL-PEG-PCL column is presented in Figure 2. As shown, the PCL-PEG-PCL column provides baseline resolutions (R > 1.5) for all of the analytes with good peak shapes within 16 min, displaying high separation performance for aliphatic and aromatic analytes ranging from nonpolar to polar nature. Except for alcohols, other analytes are eluted on the PCL-PEG-PCL column in the order of boiling points, indicating that dispersion interactions between the stationary phase and analytes play a dominant role in the separation. Moreover, the alcohols and adjacent analytes are eluted on the PCL-PEG-PCL column against the order of boiling point. Subsequent elution of alcohols can be attributed to their strong H-bonding and dipole–dipole interactions with the hydroxyl and carbonyl groups of PCL-PEG-PCL stationary phase. The halogenated benzenes also follow the order of the extent of H-bonding and dipole interactions, similar to the case of alcohols. 1,2,3-Trichlorobenzene (dipole, 2.89D) was eluted later than 1,3-dibromobenzene(dipole, 1.58D) because of its stronger H-bonding interaction with the stationary phase, though they have the same boiling point (218 °C). The above results demonstrated the high resolving capability and unique retention behaviors of the PCL-PEG-PCL stationary phase for analytes of diverse types and varying polarities. This capability was due to its unique amphiphilic structure and multiple molecular interactions involving H-bonding, dipole–dipole, and dispersion interactions.

### 2.5. Separation of Positional, Structural, and cis-/trans-Isomers

**Figure 3:**
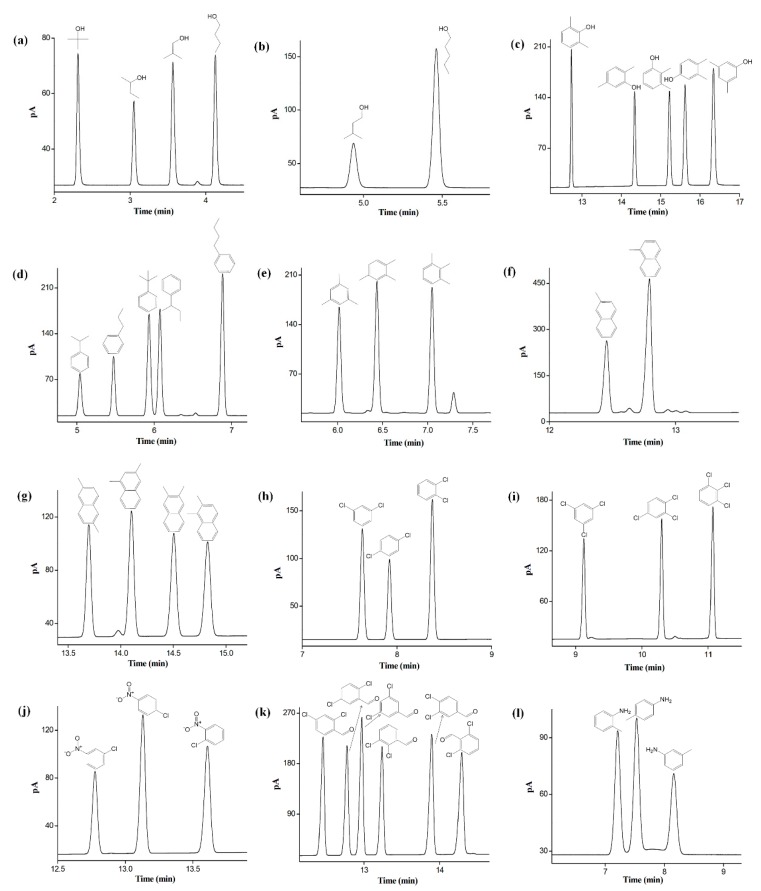
Separations of isomer mixtures of (**a**) butanol, (**b**) pentanol, (**c**) xylenol, (**d**) propylbenzene and butylbenzene, (**e**) trimethylbenzene, (**f**) methylnaphthalene, (**g**) dimethylnaphthalene, (**h**) dichlorobenzene, (**i**) trichlorobenzene, (**j**) nitrochlorobenzene, (**k**) dichlorobenzaldehyde, and (**l**) toluidine on the PCL-PEG-PCL column. Temperature program for (**a**–**k**) isomer mixtures: 40 °C (1 min) to 160 °C at 10 °C/min, flow rate at 0.6 mL/min. Temperature program for (**l**) toluidine isomers: 125 °C (10 min), flow rate at 0.6 mL/min.

High-resolution separation of isomers is of vital importance in environmental analysis and chemical industry. The separation of compounds with close natures is also a challenge in separation science. On the basis of the results described above, the PCL-PEG-PCL column was further explored for the resolving capability toward analytes of high resemblance such as positional isomers, structural isomers, and cis-/trans-isomers. Figure 3a–l presents the separation results of 12 positional and structural isomers on the PCL-PEG-PCL column, including alcohols, phenols, alkylbenzenes, alkylnaphthalenes, halogenated benzenes, benzaldehydes, and anilines. As shown, the structural and positional isomers ranging from nonpolar to polar nature achieved baseline resolutions (R > 1.5) with sharp peaks on the PCL-PEG-PCL column, the alcohols, phenols, benzaldehydes, and anilines in particular, that are liable to severe peak tailing and hard to resolve well. Figure 3a–c presents the separations of the butanol, pentanol, and xylenol isomers on the PCL-PEG-PCL column, revealing its high-resolution performance for the polar isomers. The baseline resolution of the hydroxyl group isomers indicated the contribution of H-bonding and dipole–dipole interactions to the separation process on the PCL-PEG-PCL stationary phase. As shown in Figure 3d–g, the PCL-PEG-PCL column provided good resolution for nonpolar aromatic isomers, such as propyl/butylbenzene, trimethylbenzenes, methylnaphthalenes, and dimethylnaphthalenes. The dispersioninteractions and C–H···*π* interactions between stationary phase and analytes probably played significant roles and made the PCL-PEG-PCL phase highly sensitive to the compounds of similar structures. Also, the PCL-PEG-PCL column well separated the isomers of dichlorobenzene, trichlorobenzene, nitrochlorobenzene, and dichlorobenzaldehyde (Figure 3h–k). All halogenated benzene isomers, especially the critical pairs of *m*-/*p*-dichlorobenzene and 2,4-dichlorobenzaldehyde/2,5-dichlorobenzaldehyde with boiling points difference less than 1 °C, were completely resolved, probably owing to their slightly differences in H-bonding and dipole–dipole interactions with the stationary phase. Toluidine isomers are important materials in chemical industry but they are also the known critical isomers in GC [36,37]. Besides, *o*-toluidine is listed in 24 types of carcinogenic aromatic amine by the European Union [38]. As shown in Figure 3l, the PCL-PEG-PCL column attained complete separations of the toluidine isomers (*R* = 1.83 for *o*-/*p*-toluidine, *R* = 3.39 for *p*-/*m*-toluidine). Moreover, the separation of toluidine isomers on PCL-PEG-PCL column was investigated in comparison with HP-5, DB-17, and DB-1701 commercial columns. As a result, the GC chromatograms for the separation of the toluidine isomers on PCL-PEG-PCL and commercial columns are shown in Figure S3. As shown, the PCL-PEG-PCL column baseline resolved (R > 1.5) the toluidine isomers in contrast to the overlapping or co-elution of some analytes on the HP-5, DB-17, and DB-1701 commercial columns, thereby demonstrating its advantageous resolving performance.

**Figure 4:**
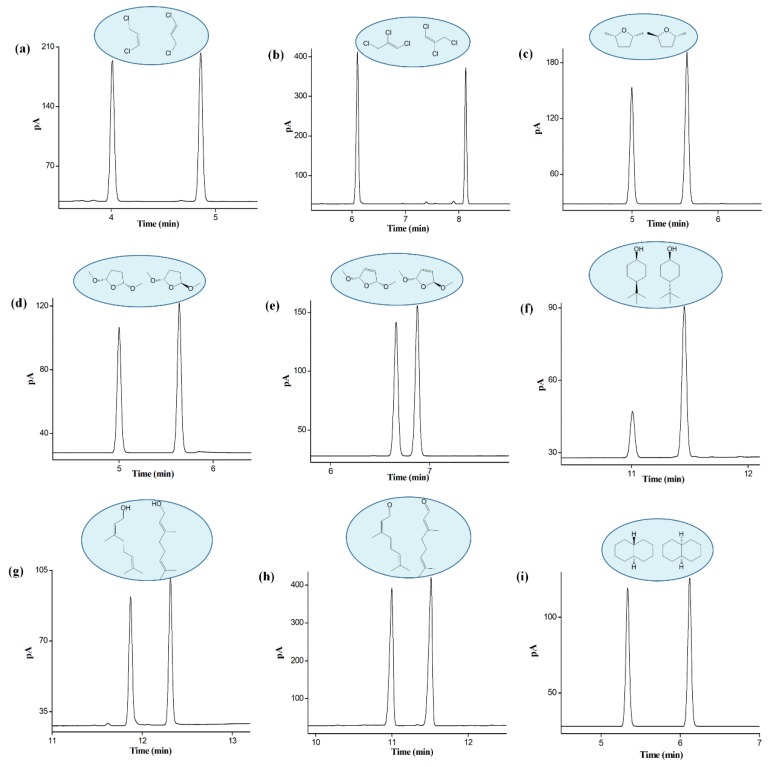
Separation of *cis*-/*trans*-isomers of (**a**) 1,3-dichloropropene, (**b**) 1,2,3-trichloropropene, (**c**) 2,5-dimethyltetrahydrofuran, (**d**)2,5-dimethoxytetrahydrofuran, (**e**) 2,5-dihydro-2,5-dimethoxyfuran, (**f**) 4-*tert*-butylcyclohexanol, (**g**) nerol/geraniol, (**h**) citral, and (**i**) decahydronaphthalene on the PCL-PEG-PCL column. Temperature program for all isomer mixtures: 40 °C (1 min) to 160 °C at 10 °C/min, flow rate at 0.6 mL/min.

Subsequently, the PCL-PEG-PCL column was further explored for its resolving capability for nine *cis*-/*trans*-isomers consisting of aliphatic and aromatic analytes. As can be seen from Figure 4a–i, the PCL-PEG-PCL column provides baseline resolutions for all *cis*-/*trans*-isomers with good peak shapes, suggesting its good distinguishing capability for analytes of high similarity. Overall, the above results demonstrate the highly selective capability of PCL-PEG-PCL stationary phase for diverse types of isomers with similar properties, which can be ascribed to its unique amphiphilic structure and multiple molecular interactions involving H-bonding, dipole–dipole, and dispersion interactions.

### 2.6. Column Operating Range 

**Figure 5:**
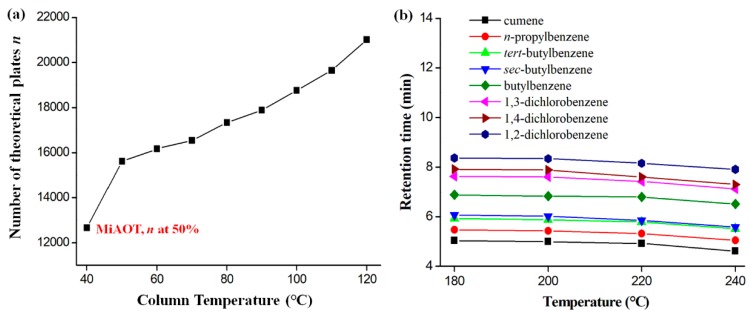
Determination of the minimum allowable operating temperature (MiAOT) was determined by naphthalene (**a**) and the thermal stability (**b**) of PCL-PEG-PCL column.

The minimum allowable operating temperature (MiAOT) was defined as the temperature where the column efficiency drops down to half of its original value at elevated temperatures [31,39]. The MiAOT of the PCL-PEG-PCL column was determined by naphthalene over atemperature range of 40–120 °C at a flow rate of 1 mL/min and the results are illustrated in Figure 5a. As shown, the column efficiency decreased gradually with temperature reduction and the half column efficiency occurred at about 40 °C, indicating its MiAOT for the GC separation. Thermal stability of the PCL-PEG-PCL column was also investigated by separation of the isomer mixtures of propylbenzene, butylbenzene, and dichlorobenzene after the column was conditioned up to each of the temperatures of 180–240 °C in 20 °C increment for 2 h, respectively. Figure 5b illustrates the effect of the conditioning temperatures on the retention times of the isomers and shows no significant variations (RSD < 2.5%) over the temperature range, suggesting its good column thermal stability up to 220 °C. For the separation of analytes with higher boiling points, PCL materials of higher thermal stability are needed to be further investigated.

### 2.7. Applications for Determination of Isomer Impurities in Real Samples

**Figure 6:**
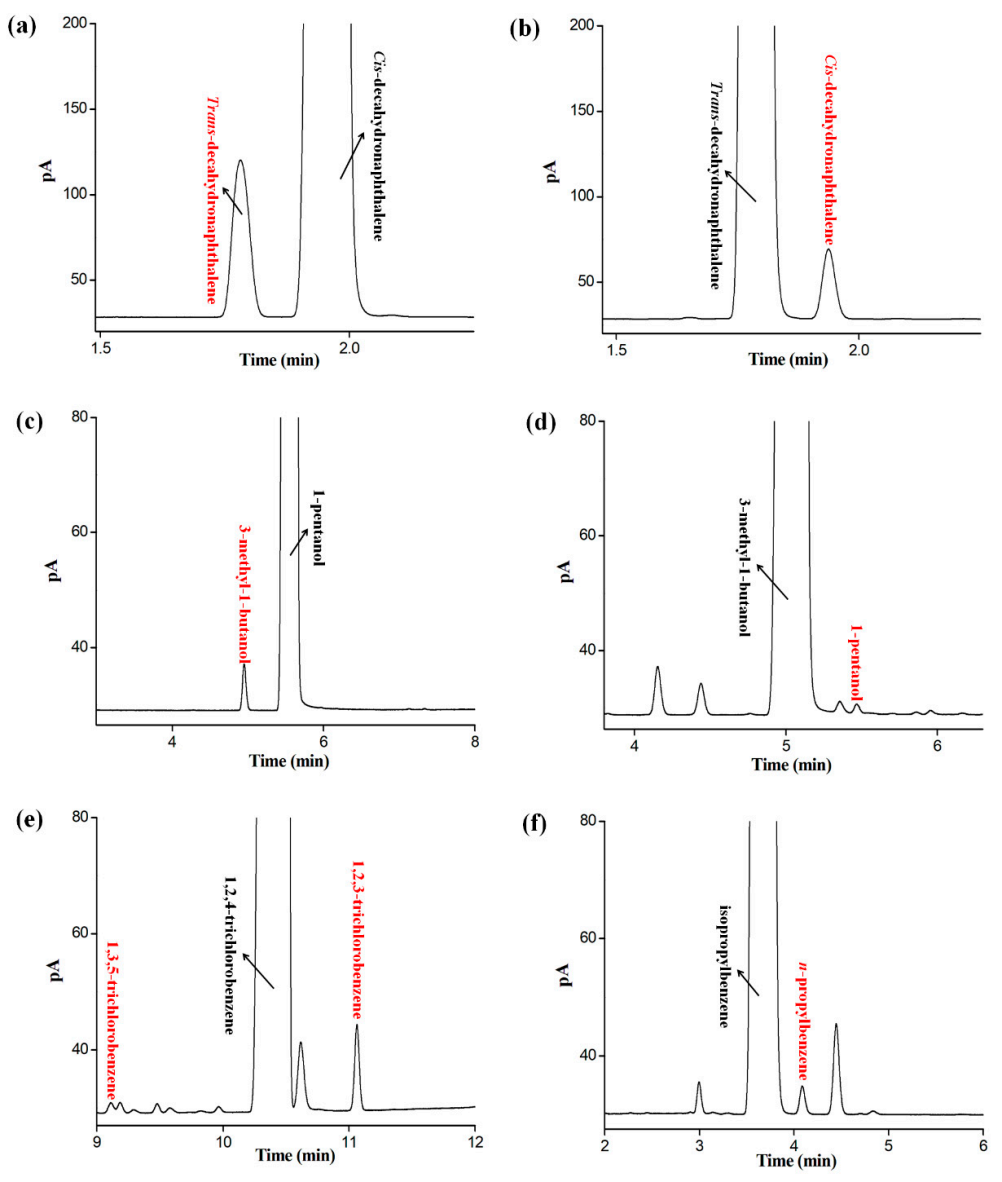
Applications of the PCL-PEG-PCL column for the determination of minor isomer impurities in the real samples of (**a**) *cis*-decahydronaphthalene, (**b**) *trans*-decahydronaphthalene, (**c**) 1-pentanol, (**d**) 3-methyl-1-butanol, (**e**) 1,2,4-trichlorobenzene, and (**f**) isopropylbenzene, respectively.

In view of the excellent separation performance of the PCL-PEG-PCL column for isomers, we applied it for the detection of isomer impurities in commercial reagent samples. Figure 6a–f provides the results for the determination of the reagent samples of *cis*-decahydronaphthalene, *trans*-decahydronaphthalene,1-pentanol,3-methyl-1-butanol,1,2,4-trichlorobenzene, and isopropylbenzene, respectively. Table 3 summarizes their content results by peak area normalization method. Except for 3-methyl-1-butanol and 1,2,4-trichlorobenzene, the measured purity of the samples was consistent with the labeled values, demonstrating the good potential of the PCL-PEG-PCL column for practical applications in GC analysis.

## 3. Experimental

### 3.1. Materials and Equipment

All the reagents and solvents employed were commercially available and were used as received without further purification. All the analytes were of analytical grade and dissolved in dichloromethane. Untreated fused-silica capillary tubing (0.25 mm, i.d.) was purchased from Yongnian Ruifeng Chromatogram Apparatus Co., Ltd. (Hebei, China). The commercial capillary columns HP-5 (10 m × 0.25 mm, i.d., 0.25 μm film thickness, 5% phenyl 95% dimethyl polysiloxane), DB-17 (30 m × 0.25 mm, i.d., 0.25 μm film thickness, 50% phenyl 50% dimethyl polysiloxane) and DB-1701 (30 m × 0.25 mm, i.d., 0.25 μm film thickness, 14% cyanopropyl phenyl 86% dimethyl polysiloxane) were purchased from Agilent Technologies and used as the reference columns.

An Agilent 7890A gas chromatograph equipped with a split/splitless injector, a flame ionization detector (FID), and an autosampler was used for GC separations. All the separations were performed under the following GC conditions: nitrogen of high purity (99.999%) as carrier gas, injection port at 300 °C, split ratio at 60:1, FID detector at 300 °C. Oven temperature programs for the GC separations were individually provided in their Figure captions. ^1^H NMR spectra were recorded on a Bruker Biospin 400 MHz instrument using tetramethyl silane (TMS) as the internal standard. IR spectra were recorded on a Bruker Platinum ART Tensor II FT-IR spectrometer. Scanning electron microscopy (SEM) images were recorded on a Zeiss Sigma 500 microscope (Zeiss, Oberkochen, Germany). Differential scanning calorimetry (DSC) measurements were performed using TA Q2000 device (TA Instruments, New Castle, DE, USA). Thermogravimetric analysis (TGA) was used on a DTG-60AH instrument (Shimadzu, Japan).

### 3.2. Synthesis of thePCL-PEG-PCLStationary Phase

**Figure 7:**
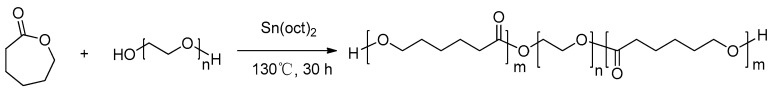
Synthesis of the PCL-PEG-PCL.

PCL-PEG-PCL was synthesized by the procedures described in Figure 7 [26]. PEG-2000 (2 g) was heated at 110 °C for 30 min in a 50 mL round-bottom flask. Then, *ε*-caprolactone (3 g) and Sn(Oct)_2_ (0.01 g) were added to the reaction vessel, the temperature was raised to 130 °C, and the mixture was stirred under nitrogen atmosphere for 30 h. The product was dissolved in chloroform (10 mL), precipitated in cold diethyl ether (20 mL), and dried under vacuum to obtain a white solid product (87% yield). m.p. 31.2–33.1 °C; ^1^H-NMR (400 MHz, CDCl_3_) δ: 4.25–4.20 (m, 2H), 4.06 (t, *J* = 6.4 Hz, 20H), 3.64 (s, 24H), 2.33 (dt, *J* = 14.8, 7.2 Hz, 24H), 1.72–1.59 (m, 48H), 1.38 (dt, *J* = 15.2, 8.0 Hz, 24H); IR (KBr, cm^−1^): 2942 (CH_2_), 2864 (CH_2_), 1721 (C=O), 1103 (C-O), 1044 (C–O).

### 3.3. Fabrication of the PCL-PEG-PCL Capillary Column

The PCL-PEG-PCL capillary column was fabricated by static coating method [40,41]. Before coating, one bare fused-silica capillary column (10 m × 0.25 mm, i.d.) was pretreated with a saturated solution of sodium chloride in methanol for the inner surface roughening of the capillary column. After the pretreatment, the column was statically coated with the solution of the PCL-PEG-PCL stationary phase in dichloromethane (0.15%, *w*/*v*) at room temperature. After the column was filled with the coating solution and sealed at one end, the solvent was evaporated at a steady speed from the other end under vacuum. At last, the column was conditioned from 40 °C to 180 °C at 1 °C/min and held at 180 °C for 7 h under nitrogen. The as-prepared PCL-PEG-PCL column was used for the following work.

## 4. Conclusions

Herein, we present the first example of employing amphiphilic block copolymer PCL-PEG-PCL as a stationary phase for capillary GC separations. Statically coated PCL-PEG-PCL column without any deactivation exhibited good separation performance and column inertness for analytes of different types with good peak shapes, including those that are labile to peak tailing. Also, the PCL-PEG-PCL stationary phase exhibited high selectivity and resolving capability for positional, structural, and cis-/trans-isomers with wide ranging polarity. The advantageous separation capability of the PCL-PEG-PCL stationary phase may derive from its unique amphiphilic structure and comprehensive molecular interactions including H-bonding, dipole–dipole, and dispersion interactions. Moreover, the PCL-PEG-PCL column was applied for the determination of minor isomer impurities in real samples, showing promise for practical analysis. This work may encourage more research and applications of amphiphilic block copolymer in separation science.

## Figures and Tables

**Table 1:** McReynolds constants of the PCL-PEG-PCL column.

Stationary Phases	X′	Y′	Z′	U′	S′	Average Polarity
*I* for PCL-PEG-PCL	876	1009	908	1098	1085	
*I* for squalane	653	590	627	652	699	
*△I* for PCL-PEG-PCL	223	419	281	446	386	351

*X′*, benzene; Y′, 1-butanol; *Z′*, 2-pentanone; U′, 1-nitropropane; S′, pyridine. Temperature: 120 °C.

**Table 2:** Abraham system constants (±s.d.) of the PCL-PEG-PCL stationary phase (*b* = 0).

T (°C)	*e*	*s*	*a*	*l*	*c*	*R* ^2^	*SE*	*F*	*n*
80	−0.001	1.326	2.239	0.425	−1.968	0.992	0.052	594	29
	(0.007)	(0.072)	(0.087)	(0.022)	(0.093)				
100	−0.009	1.108	1.991	0.400	−1.967	0.991	0.065	621	35
	(0.008)	(0.071)	(0.069)	(0.030)	(0.136)				
120	−0. 004	0.809	1.785	0.251	−1.281	0.991	0.050	543	32
	(0.007)	(0.057)	(0.070)	(0.028)	(0.125)				

*n*, Number of solutes used in the model; *R*^2^, coefficient of determination; *F*, Fisher’s factor; *SE*, standard error of the estimate.

**Table 3:** Results for the detection of isomer impurities in the commercial reagent samples on PCL-PEG-PCL column.

Samples	Labeled Purity	Measured Purity	Isomer Impurity	Content
*cis*-decahydronaphthalene	98%	98.70%	*trans*-decahydronaphthalene	1.27%
*trans*-decahydronaphthalene	98%	99.50%	*cis*-decahydronaphthalene	0.45%
1-pentanol	98%	99.44%	3-methyl-1-butanol	0.26%
3-methyl-1-butanol	98.5%	98.23%	1-pentanol	0.04%
isopropylbenzene	99%	99.31%	n-propylbenzene	0.10%
1,2,4-trichlorobenzene	99%	98.41%	1,2,3-trichlorobenzene	0.28%

## Supplementary Materials

The following are available online, Figure S1: FT-IR spectra of the PCL-PEG-PCL, Figure S2: ^1^H-NMR spectrum (CDCl_3_) of the PCL-PEG-PCL, Figure S3: GC separations of toluidine isomers on the PCL-PEG-PCL (10 m × 0.25 mm) column in comparison to the HP-5 (10 m × 0.25 mm), DB-17 (30 m × 0.25 mm), and DB-1701 (30 m × 0.25 mm) commercial columns. Table S1: Solutes and their descriptors for determining the system constants of the stationary phases by the Abraham solvation parameter model.
