# Supplementary material for: Amphiphilic Block Copolymer PCL-PEG-PCL as Stationary Phase for Capillary Gas Chromatographic Separations
Source: Molecules. 2019 Aug 30;24(17):3158. doi: 10.3390/molecules24173158 (PMC6749289; doi:10.3390/molecules24173158)
Supplement: Supplementary file 1 [file molecules-24-03158-s001.pdf]

**Supplementary materials**

# Amphiphilic Block Copolymer PCL-PEG-PCL as Stationary Phase for Capillary Gas Chromatographic Separations

**Tao Sun [a,*], Xiaomin Shuai [b], Kaixin Ren [a], Xingxing Jiang [a], Yujie Chen [a], Xinyu Zhao [a], Qianqian Song [a], Shaoqiang Hu [a] and Zhiqiang Cai [b,*]**

[a] College of Chemistry and Chemical Engineering, Henan Key Laboratory of Function-Oriented Porous Materials, Luoyang Normal University, Luoyang 471934, P. R. China

[b] Liaoning Province Engineering Research Center for Fine Chemical Engineering of Aromatics Downstream, School of Petrochemical Engineering, Shenyang University of Technology, Liaoyang, 111003, Liaoning, P. R. China

* Correspondence: suntao2226@163.com (T.S.); czq0601@sut.edu.cn (Z.C.)

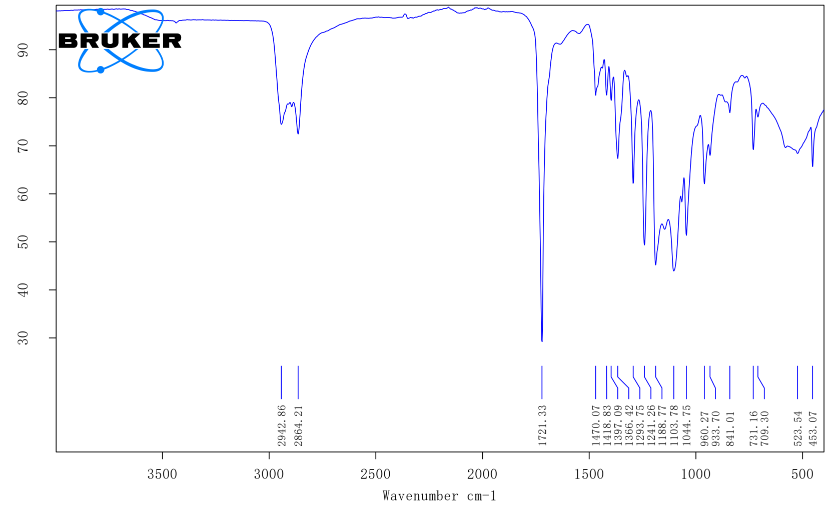

**Fig. S1.** FT-IR spectra of the PCL-PEG-PCL

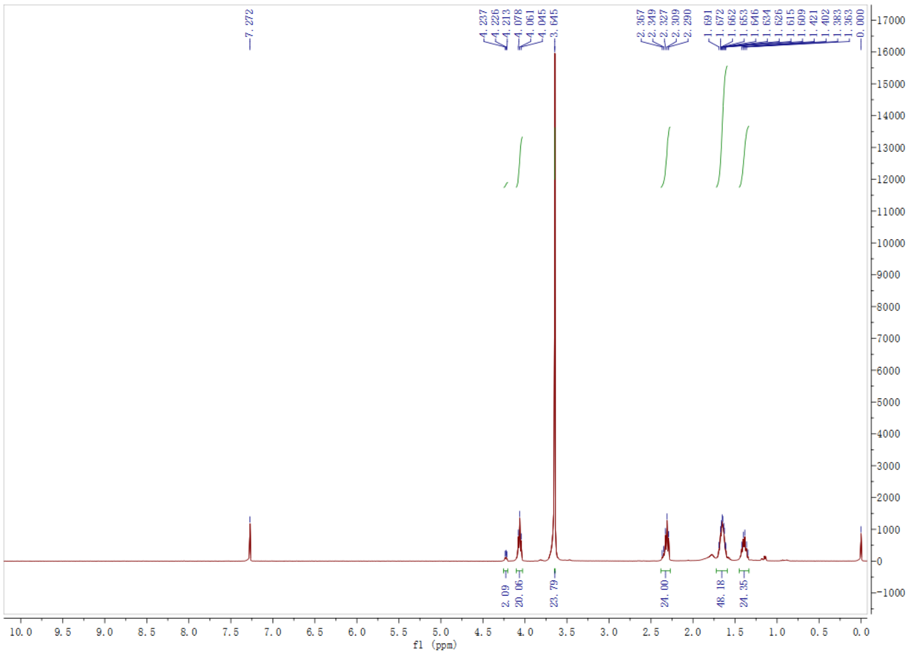

**Fig. S2.** $^{1}$H-NMR spectrum ($CDCl_3$) of the PCL-PEG-PCL

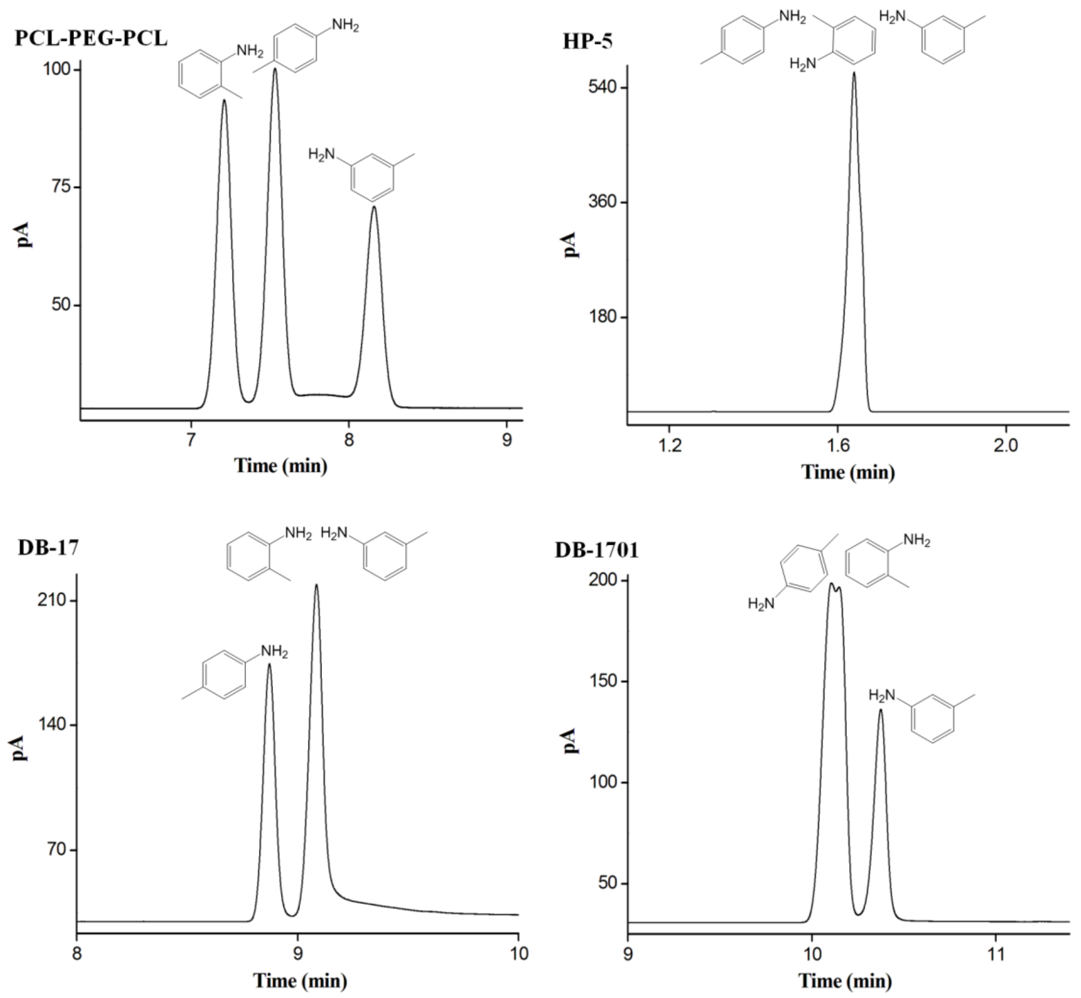


**Fig. S3**. GC separations of toluidine isomers on the PCL-PEG-PCL (10 m × 0.25 mm) column in comparison to the HP-5 (10 m × 0.25 mm), DB-17 (30 m × 0.25 mm), and DB-1701 (30 m × 0.25 mm) commercial columns. The GC separation on four columns was performed under same condition. Temperature programs for toluidine: 125℃, flow rate at 0.6 mL/min.

**Table S1** Solutes and their descriptors for determining the system constants of the stationary phases by the Abraham solvation parameter model.

| Probe solute | E | S | A | B | L |
|---|---|---|---|---|---|
| Methyl hexanoate | 0.080 | 0.60 | 0 | 0.45 | 3.874 |
| Methyl heptanoate | 0.072 | 0.60 | 0 | 0.45 | 4.392 |
| Methyl octanoate | 0.065 | 0.60 | 0 | 0.45 | 4.838 |
| 1-Pentanol | 0.219 | 0.42 | 0.37 | 0.48 | 3.106 |
| 1-Hexanol | 0.210 | 0.42 | 0.37 | 0.48 | 3.610 |
| 1-Heptanol | 0.211 | 0.42 | 0.37 | 0.48 | 4.115 |
| 2-Pentanone | 0.143 | 0.68 | 0 | 0.51 | 2.755 |
| 2-Hexanone | 0.136 | 0.68 | 0 | 0.51 | 3.262 |
| 2-Heptanone | 0.123 | 0.68 | 0 | 0.51 | 3.760 |
| 2-Octanone | 0.108 | 0.68 | 0 | 0.51 | 4.257 |
| 1-Bromopentane | 0.356 | 0.40 | 0 | 0.12 | 3.611 |
| 1-Bromohexane | 0.349 | 0.40 | 0 | 0.12 | 4.130 |

| | | | | | |
|---|---|---|---|---|---|
| 1-Bromoheptane | 0.343 | 0.40 | 0 | 0.12 | 4.663 |
| 1-Bromooctane | 0.339 | 0.40 | 0 | 0.12 | 5.090 |
| 1,2,3-Trichlorobenzene | 1.03 | 0.86 | 0 | 0 | 5.419 |
| 1,2,4-Trichlorobenzene | 0.98 | 0.81 | 0 | 0 | 5.248 |
| 1,3,5-Trichlorobenzene | 0.98 | 0.73 | 0 | 0 | 5.045 |
| 1,2-Dichlorobenzene | 0.872 | 0.78 | 0 | 0.04 | 4.518 |
| 1,3-Dichlorobenzene | 0.847 | 0.73 | 0 | 0.02 | 4.410 |
| 1,4-Dichlorobenzene | 0.825 | 0.75 | 0 | 0.02 | 4.435 |
| Toluene | 0.601 | 0.52 | 0 | 0.14 | 3.325 |
| Ethylbenzene | 0.613 | 0.51 | 0 | 0.15 | 3.778 |
| *n*-propylbenzene | 0.604 | 0.50 | 0 | 0.15 | 4.230 |
| *n*-Butylbenzene | 0.600 | 0.51 | 0 | 0.15 | 4.730 |
| 1,2,3-Trimethylbenzene | 0.728 | 0.61 | 0 | 0.19 | 4.565 |
| 1,2,4-Trimethylbenzene | 0.677 | 0.56 | 0 | 0.19 | 4.441 |
| 1,3,5-Trimethylbenzene | 0.649 | 0.52 | 0 | 0.19 | 4.344 |
| 2,6-Dimethylaniline | 0.972 | 0.89 | 0.2 | 0.46 | 5.028 |
| 2,3-Dimethylphenol | 0.85 | 0.81 | 0.53 | 0.36 | 4.952 |
| 2,4-Dimethylphenol | 0.843 | 0.80 | 0.53 | 0.39 | 4.770 |
| 3,4-Dimethylphenol | 0.83 | 0.86 | 0.56 | 0.39 | 4.980 |
| 3,5-Dimethylphenol | 0.82 | 0.84 | 0.57 | 0.36 | 4.856 |
| *o*-Toluidine | 0.966 | 0.92 | 0.23 | 0.45 | 4.442 |
| *m*-Toluidine | 0.946 | 0.95 | 0.23 | 0.45 | 4.463 |
| *p*-Toluidine | 0.923 | 0.95 | 0.23 | 0.45 | 4.452 |
| 2-Chloroaniline | 1.033 | 0.92 | 0.25 | 0.31 | 4.674 |
| 3-Chloroaniline | 1.053 | 1.10 | 0.30 | 0.30 | 4.909 |
| 4-Chloroaniline | 1.060 | 1.13 | 0.30 | 0.32 | 4.889 |
| Benzene | 0.608 | 0.53 | 0 | 0.14 | 2.760 |
| Acetophenone | 0.818 | 1.01 | 0 | 0.48 | 4.501 |
| Phenol | 0.805 | 0.89 | 0.6 | 0.31 | 3.766 |
| Benzaldehyde | 0.820 | 1 | 0 | 0.39 | 4.008 |
